# Managing perturbations during handover meetings: a joint activity framework

**DOI:** 10.1002/nop2.29

**Published:** 2015-09-24

**Authors:** Eric Mayor, Adrian Bangerter

**Affiliations:** ^1^ University of NeuchâtelInstitute of Work and Organizational PsychologyNeuchâtelSwitzerland

**Keywords:** Handoff, handover, interpersonal communication, nurses, work organization

## Abstract

**Aim:**

To document the prevalence of perturbations of handover meetings and understand how nurses manage temporal, physical and social meeting boundaries in response to perturbations.

**Background:**

Handovers are joint activities performed collaboratively by participating nurses. Perturbations of handover are frequent and may potentially threaten continuity of care.

**Design:**

We observed and videotaped handovers during five successive days in four nursing care units in two Swiss hospitals in 2009.

**Methods:**

Videorecordings were transcribed. All perturbations during the handovers were noted. We performed content analysis of the sources of perturbations from the notes and interactional micro‐analyses of handover interactions based on video and transcripts.

**Results:**

Nurses are the most frequent sources of perturbations during handovers. Perturbations are collaboratively managed. A tacit division of labour is enacted via multimodal communication strategies, whereby perturbations are dealt with using both linguistic and bodily signals.

## Introduction

Nursing handover meetings ensure transmission of patient information and responsibility between shifts of caregivers. They also serve functions like interpreting experience or maintaining unit cohesion (Grosjean & Lacoste 1999, Bangerter *et al*. 2011, Mayor *et al*. 2012). They are an essential part of hospital work coordination. Given their importance (Staggers & Blaz 2013), efforts have focused on identifying problematic aspects and improving them (Alvarado *et al*. 2006, Arora *et al*. 2009). Well‐studied areas include efficiency (e.g. Sherlock 1995), content (e.g. Mayor *et al*. 2012) and functions of handovers (e.g. Kerr 2002). One problem that has been little studied concerns the frequent *perturbations* of handover (Meissner *et al*. 2007). Perturbations are events that impinge on handover meetings (often involving solicitations of one or several handover participants). They emerge from a work environment featuring recurrent multitasking (Kalisch & Aebersold 2010) or lack of dedicated space (Arora *et al*. 2009) and may lead to an interruption of the handover activity if not managed appropriately. Handover participants deploy collaborative actions to keep perturbations from becoming interruptions. Research is needed to better understand the complexities of perturbations and develop strategies to manage them (Kalisch & Aebersold 2010, Hopkinson & Jennings 2013).

Here, we investigate how nurses manage perturbations of shift handover meetings, using a theory of joint activity (Clark 1996, 1999, 2006) as an analytical framework. Joint activities are focused undertakings by groups of individuals. They need to be protected from competing activities, i.e. their temporal, physical and social boundaries need to be managed. We document two aspects of boundary management in handover meetings: division of labour and multimodality. Division of labour (Clark 1996) means that handover participants can collaborate in an asymmetrical way to maintain focused activity, by adopting different roles. For example, a side participant in handover (e.g. an outgoing nurse who is not delivering a report) could be designated in advance to respond to perturbations, thereby allowing her colleagues to continue with the handover. Multimodality (Stivers & Sidnell 2005) means the intertwined use of linguistic and non‐linguistic (gaze, gesture) communicative signals. Resources drawn on by participants in collaborative tasks are not only verbal in nature. For example, in medical consultations, patients may describe symptoms via words, gestures or facial mimicry (Heath 2002). Much research exists on the multimodal coordination of joint activities in medical settings (Heath 1986, Mondada 2014). But, multimodal interactional analyses of handover meetings (Grosjean 2004) are rare. Studying handover as a joint activity shifts the focus away from caregivers as passive receivers of interruptions and allows understanding how they actively and collaboratively shape the handover meeting. It opens up the possibility of conceptualizing boundary management as a visible aspect of nursing work.

### Background

#### Joint activities

Joint activities are interrelated verbal or bodily actions performed by several people with the shared understanding that they are performing their individual actions as part of a whole (Clark 1996, 2006). They typically involve a joint focus of attention. Examples include playing a musical duet, navigating a sailboat, or talking together. Joint activities are coordinated via language and nonverbal actions (e.g. gestures) (Clark 1999). Participants coordinate five main aspects (Clark 2006): participants, content (what the activity is about) roles (what each participant will do), timing (when the activity and its component actions will be performed) and location (where they will be performed). In everyday joint activities, these agreements are entered into step by step and constitute joint commitments that participants expect each other to honour (see Bangerter & Mayor 2013).

People often pursue several goals at any given time. Thus, performing a joint activity entails suspending other activities. As time goes by, those activities impinge on the joint activity, threatening its integrity. Accordingly, joint activities need to be protected by participants. This entails managing their temporal, physical and social boundaries. Managing temporal boundaries (Bardram 2000) involves agreeing to enter into the activity or exit from it. In face‐to‐face interactions, physical boundaries are defined by how participants position themselves (Kendon 1990). Gaze and posture also mark social boundaries (Goffman 1971) of a joint activity: who is part of it (Clark 2005) or not. Social boundaries of joint activities are actively preserved against intrusions (Goffman 1971). Arriving individuals are sensitive to this and announce their arrival to request attention and permission to enter into the ongoing activity (Pillet‐Shore 2008). Participants may grant or refuse access. Granting access may involve summarizing the previous activity for the newcomer to facilitate their joining. Refusing access may be done more subtly, e.g. not rearranging the spatial configuration of bodies to incorporate the newcomer, averting gaze or not summarizing the previous activity (Pillet‐Shore 2010).

#### Studying handover perturbations as joint activity

Handover meetings are a special case of joint activity. They occur among multiple parallel activities that constitute hospital work (Ren *et al*. 2008). In recurrent work routines in organizations, coordination problems that spontaneously emerging joint activities pose are often preempted by convention. Handovers are often preprogrammed at a fixed time in a fixed room with a fixed group of participants and communication embodies norms, shared practices and culture of the individual unit (Grosjean & Lacoste 1999). However, on‐going coordination activity remains necessary to orchestrate four aspects of handover boundary management: Beginning the activity, maintaining it in the face of perturbations, suspending and reinstating it to deal with unavoidable perturbations and ending it. This study aimed to understand the frequency and the sources of perturbations during shift handovers in four nursing units. In addition, we aimed to analyse in detail the collaborative management of perturbations in one selected nursing unit.

## The Study

### Design

In a field study conducted in 2009, we observed and video‐taped shift handover meetings in four nursing units (five consecutive days in each unit). Our findings are grounded in content analysis and detailed interactional analyses.

### Sample

We studied two surgery units and two intensive care units (ICUs). We studied one unit of each type in a public hospital and one of each type in a private hospital (private hospitals may have different kinds of patients and different resources; studying both types of hospitals may improve the generalizability of the findings). The hospitals had participated in an earlier study (Mayor *et al*. 2012). We first contacted the nursing directors, explained the study goals and obtained access for on‐site observation and video‐recordings of handover meetings. We then contacted the head nurses of selected units and explained our project. We then presented the study to team members in a meeting. Posters were also displayed in the units to explain the study procedures. Unit capacity varied from 5–28 beds. Between 18–29 nurses work in each unit. Work is usually organized in shifts of 12 hours.

### Data collection

A video camera fixed on a tripod recorded handover and an observer (the first author) filled out an observational grid describing the perturbation (who, what) and its time of occurrence. The grid also served to document identity and location of participants to facilitate speaker identification. Table 1 below displays the number of handovers and personnel present (mean and SD) in each of the units. Technical problems rendered recordings of one handover in each ICU unusable. All handovers were verbal and were supported by written documentation (paper patient files and personal notes).

**Table 1:** Number of handovers and average number of persons present for handovers by unit

	Surgery public	Surgery private	ICU public	ICU private
Number handovers	15	10	10	10
Average persons (sd)	7·3 (2·9)	8·4 (1·5)	6·1 (1·6)	4·9 (1·7)

### Ethics

We obtained consent from participants in a meeting we organized in each unit. We informed participants of each unit of the period during which data collection would take place. Anonymity was guaranteed and participants agreed to publication of anonymized snapshots from videos and excerpts of talk. No particular ethical issue was identified.

### Data preparation, coding and analysis

We computed the frequency of perturbations from the grid. A perturbation is an event that can potentially lead to a breakdown of focused talk in the handover. We also coded the source of each perturbation as either ‘nursing personnel’, ‘physicians’, ‘telephone’, ‘patients’, ‘mixed groups’ (nurses and physicians) or ‘other’. Each video recording was transcribed word‐for‐word.

### Rigour

We checked interrater agreement for coding of perturbation sources. Agreement between two coders was excellent (*kappa* = 0·86, double coding of 40% of data). For qualitative analyses, we focused on transcripts and videotapes of one care unit, the public hospital surgery unit. Transcripts were subjected to multiple readings by the first author and potentially interesting cases of boundary management were discussed, often by returning to the videotape. We then selected several cases for qualitative micro‐analysis based on how emblematic they are of different aspects of boundary management. These cases were then transcribed in greater detail, with particular attention to multimodal aspects (transcription conventions in Appendix A) and translated into English.

## Findings

### Analysis of perturbations

**Figure 1:**
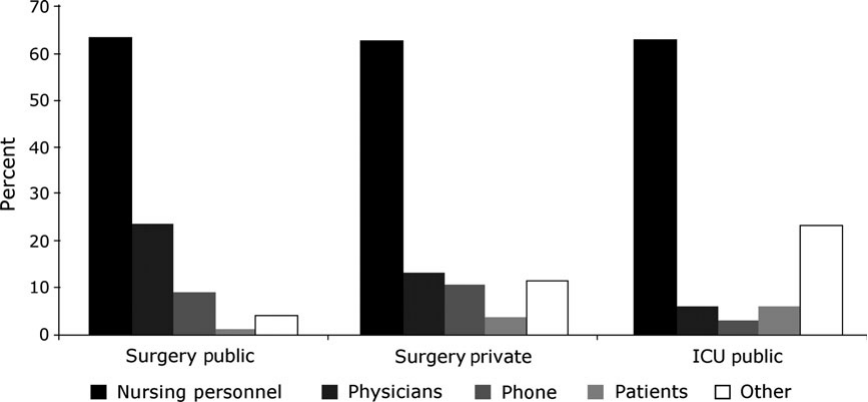
Distribution of sources of perturbation (percentages add to 100% in each unit).

In the public hospital, there were 103 perturbations in the surgery unit (during 340 minutes, 1 perturbation every 3·30 minutes on average) and 37 in the ICU (during 90 minutes, 1 perturbation every 2·43 minutes on average). In the private hospital, there were 117 perturbations in the surgery unit (during 390 minutes, 1 perturbation every 3·33 minutes on average) and only 1 perturbation in the ICU during 78 minutes. Thus, all units except the private hospital ICU evidenced roughly one perturbation every 3 minutes on average. This unit had developed a preventive strategy to manage perturbations: Handovers occurred in a small break room rather than at the nursing station. Some nurses from the outgoing shift who were not responsible for the handover were posted outside the room and took care of perturbations. Participants were sure that no perturbation would occur and thus dispatched in the entire space of the room. The three other units dealt with perturbations in an ad hoc manner. Figure 1 depicts the sources of perturbations in these units. In each unit, nurses are the most frequent sources, accounting for almost two‐thirds of perturbations, followed by physicians. Phone calls were more frequent in both surgery units than in the ICU. Perturbations caused by patients were rare in all units. Other sources (e.g. staff cleaning the floor) were rare in the surgery unit of the public hospital, but somewhat more frequent in the surgery unit of the private hospital and even more so in the ICU.

In sum, if not preempted (as in the private hospital ICU), perturbations are common in shift handover meetings. They are mainly caused by members of the nursing team themselves. This analysis is suggestive of the extent to which competing activities impinge on the handover. We now turn to a qualitative microanalysis of how participants deal with these competing activities, via ad hoc management of handover boundaries.

### Boundary management strategies

We examine boundary management in beginning handover, maintaining handover in the face of perturbations, marginalizing non‐ratified participants in the face of repeated perturbations and ending handover.

#### Beginning handover

Handover meetings emerge from a pre‐handover phase (Grosjean & Lacoste 1999), as not all participants arrive in the nursing room at the same time. Some nurses are present in the room prior to the meeting to accomplish administrative tasks like updating patient files. This essentially solitary activity may lead to sporadic discussion. The handover begins with a participant (outgoing or incoming) attempting to focus attention by declaring the beginning of the meeting.

Excerpt 1 shows how the handover gets initiated among ongoing multifocal pre‐handover activity. It is scheduled to start at 7 AM. The first nurse to arrive is an outgoing nurse, Paul, who is working on a patient file when the recording begins. He is joined by the other outgoing nurse, Pam. They discuss some patients and then continue to update their files. A moment later, Pam complains that no one is coming for the handover. Paul agrees. For approximately 5 minutes, nurses and physicians enter and exit the room, discussing various issues and manipulating documents. The nurses then wait for a colleague who is late while performing other activities (e.g. consulting patient files). The handover is initiated by Ema and Paul.

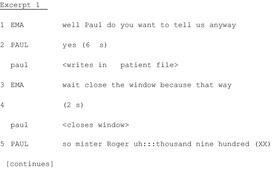

At Line 1, Ema asks Paul to start ‘anyway’, i.e. although not all participants are present and Paul complies, ‘yes’. From Ema's request and Paul's uptake, the official activity is now doing the handover. This moment marks the beginning of a transition from simultaneous parallel activities to a focused joint activity. However, Paul does not start immediately, but first finishes his own activity (writing in a patient file). Moreover, the beginning is again postponed by the action of closing the window (to reduce noise from the street), aimed at protecting the meeting from a potential perturbation. Beginning handover is an effortful process featuring progressive suspension of individual activities and establishment of a joint focus of attention.

#### Maintaining handovers in the face of perturbations

Participants try to maintain handover talk in the face of persistent perturbations. They collaborate to minimize the impact of the perturbation on the main track of talk (i.e. the report by the outgoing nurse). This often involves: (1) division of labour in: the team, with peripheral participants spontaneously accomplishing such tasks; and (2) multimodal communication, like gaze and gestures to exchange information and objects without disrupting the main line of talk. In Excerpt 3, the outgoing nurse Pam is discussing a patient when the phone rings, attracting attention and threatening the integrity of the joint activity.

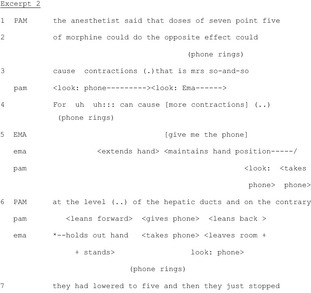

In Lines 1‐3, Pam is explaining why morphine has not been administered to a patient. This explanation continues throughout the excerpt. At the end of Line 2, the phone rings. Pam immediately notices the perturbation, orienting her gaze towards it at the beginning of Line 3. Meanwhile, she continues talking. Only at the end of her utterance does she glance at the caller identification screen to announce the caller. At Line 4, Pam continues her explanation by recycling (Schegloff 1987) part of her previous utterance. This is accompanied by another ring and Ema's request for the phone which she performs both verbally (in overlap to Pam's talk) and bodily (Line 5). At the end of Line 5, after Ema's request, Pam looks at the phone again and takes it, still talking about the patient. At Line 6, the nurses exchange the phone and Ema leaves the room while Pam returns to her original position.

Pam and Ema's coordinated actions serve to minimize the impact of the perturbation by transporting its source outside the room. By exploiting multimodal resources (gaze, gestures, exchange of objects) and an ad hoc division of labour according to their organizational roles during handover, they simultaneously perform two joint activities, handover talk and removing the phone, thereby maintaining the integrity of the handover in the face of the perturbation.

The next excerpt illustrates division of labour when dealing with a perturbation initiated by a non‐ratified participant (an aide, Amy). A peripheral participant, the outgoing nurse Pam (not currently giving the report), coordinates with Amy without producing a suspension of the handover. The other outgoing nurse, Deb, is reporting on a patient when Amy enters.

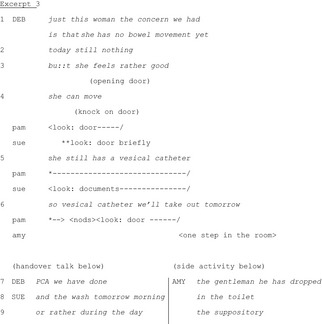

At Lines 1‐3, Deb is discussing a patient. On Line 3, someone opens the door off camera. Pam starts gazing towards the door (beginning of Line 4). She maintains this gaze direction throughout the excerpt, thus displaying availability to an interaction with the person entering. Amy then knocks (Line 4). Sue briefly looks at the door. She then rapidly refocuses on her documents, thereby displaying unavailability to a potential interaction at that point. At Line 5, Deb starts discussing the patient's equipment. This is a new topic and this moment would have been appropriate for dealing with Amy's entry (Chevalley & Bangerter 2010), but instead she proceeds with the handover. Note that Amy does not initiate her request at this moment either, thus showing deference to the handover in course. At Line 6, Pam nods while still looking at Amy, simultaneously to Deb's continuing her report. Amy treats the nod as an invitation to proceed with her request, which she does simultaneously to ongoing handover talk. Here again, two joint activities are simultaneously performed: the handover and managing Amy's request. It was not essential for Pam to listen to Deb's report as she was leaving the unit. She thus spontaneously and nonverbally responded to Amy's request, extracting herself from the boundaries of the handover while Deb and Sue continued. Again, a tacit division of labour supported by multimodal signals (gaze, nods) served to protect the integrity of the handover.

Sometimes suspending handover is unavoidable to deal with unexpected perturbations that cannot be minimized. Nurses often first look at the source of the perturbation. A brief look serves to discourage further perturbation (by manifesting unavailability), whereas a longer look constitutes an invitation to explain the reason of the perturbation (by manifesting availability). When the activity restarts, some nurses do not refocus on the handover. Hence, perturbations can lead nurses to exit the handover.

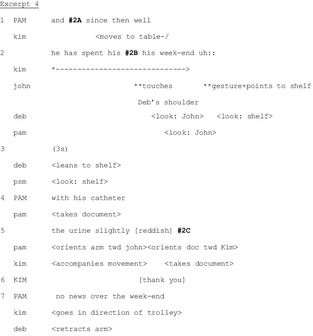

**Figure 2:**
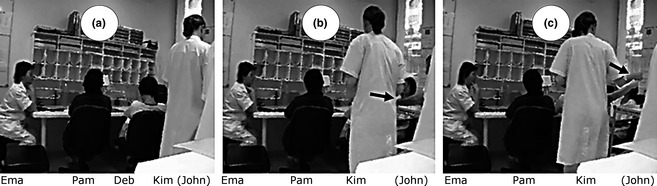
Bodily request of a document. (a) Initial position of participants. (b) John touches Deb's shoulder (see arrow) and Kim moves to the table. (c) Kim accompanies Deb's arm movement and takes the document (see arrow).

At Lines 1‐2, Pam starts discussing a patient's equipment. At the middle of Line 1, a junior physician, Kim, who is near John (Figure 2a), walks to the table where the nurses are seated and stops at Line 2, after John touches Deb's shoulder (Figure 2b). Doing so, he recruits Deb's attention and, certainly unintentionally, Pam's. Pam suspends her speech and produces a disfluency, *uh*::. During this, John nonverbally requests that Deb pass him a document from the shelf behind the table. He does this by: (a) first looking at her; (b) tracing a square with both index fingers while maintaining mutual gaze; and (c) pointing towards a spot on the shelf. This is followed by a 3‐second pause in the handover (Line 3), during which Deb leans towards the shelf and Pam looks at the shelf. Pam then continues the handover (Line 4: *with his catheter*) and Deb takes the document. At Line 5, Deb moves her arms in John's direction. Kim accompanies her gesture with her own arm. Deb apparently understands this as a request for the document as she ends up giving it to Kim and not John (Figure 2c). Kim takes the document and thanks Deb (Line 5) in overlap with Pam's talk. Deb returns to her initial posture and Kim returns to John.

In this excerpt, John entered the boundary of the handover by touching a peripheral participant, as did Kim later by attempting to help in the transfer of the document. John's gesture recruited Deb's attention but also Pam's (which is probably precisely what John was trying to avoid). This produced a short breach in handover talk, despite participants' efforts.

#### Marginalization strategies against repeated perturbations

Physicians sometimes attend handover, although this is not routine. They also sometimes ask questions during the handover. Nurses respond briefly and the handover usually continues without further interventions. In contrast, in the following excerpts, a physician, Tim, repeatedly asks questions, suggesting he considers himself entitled to participate in the handover (Goffman 1971). This construal is not shared by the nurses, who marginalize him using strategies like not looking at him when he speaks and delaying answering his questions. Excerpts 5a and 5b contrast the nurses' cooperative treatment of Tim's first question (which is tolerable as an exception) with the uncooperative treatment of subsequent questions. Excerpt 5a represents the usual treatment of a physician's question, whereas in Excerpt 5b, the nurses act to preserve the social boundaries of the joint activity repeatedly impinged on by Tim. Before Excerpt 5a, the outgoing nurse Sue has started reporting on a patient.

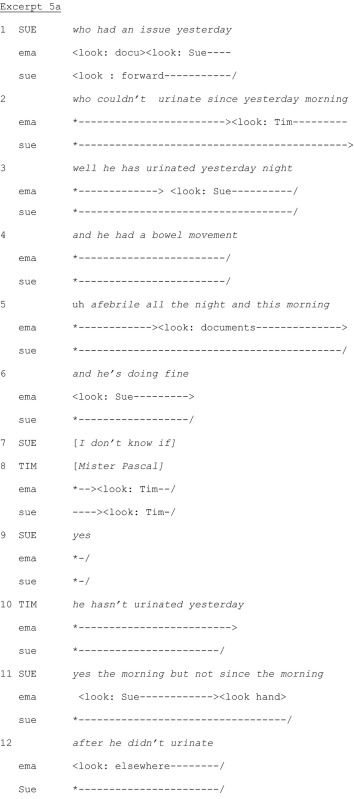

On Lines 1‐6, Sue is describing the patient's inability to urinate (which has resolved itself) and that he had a bowel movement. She continues on Line 7 when Tim utters the patient's name with a rising intonation on Line 8, displaying he has been listening to the handover. Sue confirms (Line 9) it is this patient she is talking about. Tim then asks for confirmation about the patient's urination, revealing his misunderstanding of Sue's explanations. Sue clarifies on Lines 11 and 12. There is no delay in Sue's answers.

Excerpt 5b occurs approximately 24 turns of talk after Excerpt 5a. Tim has repeatedly asked questions and made observations. Excerpt 5b starts with Sue discussing a patient.

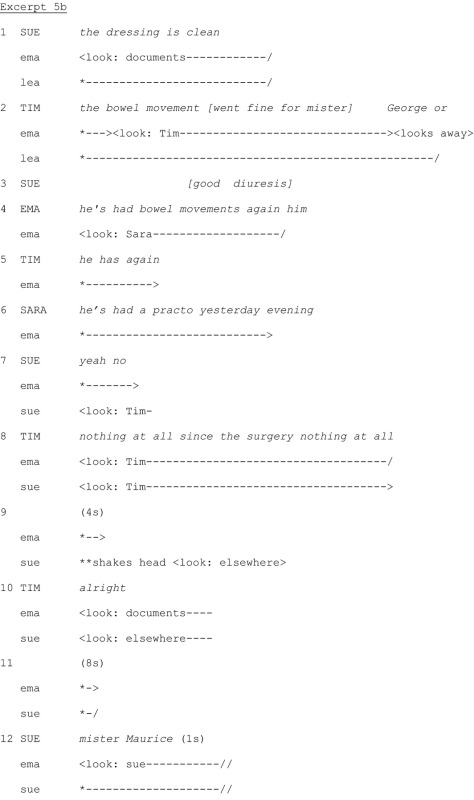

On Line 1, Sue discusses the state of the patient's dressing, which she assesses as clean. After a two‐second pause in Sue's report, Tim again self‐selects as a ratified participant and asks whether the patient has had bowel movements (Line 2). On Line 3, Sue continues, overlapping with Tim's turn of talk. She does not answer Tim's question and doesn't look at him. By talking in overlap, long after the beginning of Tim's statement (and despite Tim's higher status), she acts as if the physician's question did not occur, displaying her construal of Tim's question as an invasion of the handover. On Line 4, Ema, the head nurse, also asks whether the patient has had bowel movements, thereby respecifying the physician's question. She selects Sara, not Sue, as next speaker by looking at pher. Ema asking about the bowel movements also legitimizes Tim's question, which he reiterates on Line 5. Sara states which drug the patient received the previous evening on Line 6, which addresses the patient's constipation issue, but doesn't answer the question. Sue finally answers Tim's question (‘yeah no’) on Line 7 and starts looking at him. The delayed placement of her answer shows her disalignment with Tim. Tim repeats the question twice on Line 8, manifesting his construal of Sue's answer as unclear. On Line 9, Sue shakes her head and looks elsewhere. By withdrawing gaze, she displays lack of further interest in interacting with Tim. After a pause, Tim says *well* (Line 10), thus acknowledging the end of the interaction. Ema simultaneously starts looking at her documents, reorienting to the handover. After another long pause (8 seconds), Sue starts discussing another patient on Line 12.

There is a marked contrast between treatments of Tim's initial and subsequent questions: Sue participated in the exchange started by Tim's initial question in Excerpt 5a. She looked at him throughout and answered his question promptly. But in Excerpt 5b, she displays reluctance to answer the question by not looking at him for several turns of talk, speaking in overlap long after the onset of his turn, delaying her answer, providing information only nonverbally and withdrawing gaze rapidly, thus manifesting unavailability to interact with Tim and orientation to the handover as a joint activity between nurses only.

#### Ending handover

In our data, ending handover is often explicitly signalled by expressions like ‘that's it’ or expressions of gratitude (e.g. incoming nurses thanking the outgoing nurse for the report). This is followed by a return to multiple individual or pairwise activities in the nursing room (e.g. preparing medication or further talk about patients or other topics). Excerpt 2 illustrates such an ending. It starts with the end of the discussion of the last patient.

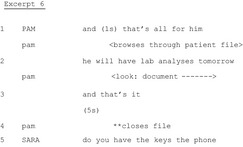

After reporting on a patient, Pam initiates the closing of the patient's report on Line 1 (*and that's all for him*) while browsing through the patient file. Next, she mentions laboratory analyses to be performed the day after, reading from the file (Line 2). She then initiates the closing of the meeting (*and that's it*) and closes the file (Line 4). The ending is followed by a five‐second pause after which another nurse, Sara, asks the incoming nurse Deb whether she has the keys and phone. This exchange continues after the excerpt. Simultaneously, another nurse gets up, goes to the sink and washes her hands, preparing for clinical activities.

Ending handover needs to be done explicitly and constitutes an action distinct from the last topic (Bangerter & Clark 2003). Explicitly marking the end of the handover is functionally similar to agreeing to begin the handover (Excerpt 1). Both actions serve to coordinate the temporal boundaries of the joint activity (Grosjean & Lacoste 1999, Mondada 2006), separating it from other preceding and following activities that also involve verbal interactions.

## Discussion

In a field study of handover meetings in nursing units, we examined the frequency of perturbations, their sources and how handover participants maintain the temporal, physical and social boundaries necessary to protect the integrity of handover. Perturbations were as frequent or more frequent than interruptions documented in previous studies (Kalisch & Aebersold 2010), except in one unit where they were preempted by gatekeepers. The routine, planned nature of handover meetings does not afford any particular protection from perturbations. Nursing personnel are the most frequent sources of perturbations, which they cause by entering and exiting the handover room and soliciting handover participants. These perturbations illustrate the complexity of hospital work, which is composed of multiple parallel activities (Fussell *et al*. 2004) that repeatedly impinge on the handover. Participants collaboratively manage the boundaries of handover meetings to minimize the impact of these perturbations.

Perturbations are managed via a tacit division of labour according to nurses' temporal status as incoming or outgoing. As outgoing nurses who are not giving the report will subsequently leave, they are thus more available to deal with perturbations. Incoming nurses need to process information discussed during the handover, but can deal with perturbations to some extent because their colleagues can still repeat information to them after the handover. Outgoing nurses giving the report are least likely to handle perturbations.

This tacit division of labour is often enacted via multimodal communication strategies, whereby perturbations are dealt with using both linguistic and bodily signals. Multimodal communication enables several parallel processes, including attracting attention of specific participants without jeopardizing talk, acknowledging perturbations as legitimate, transporting perturbations outside the handover setting, or disregarding non‐ratified participants.

Nurses are dissatisfied by handover perturbations (Meissner *et al*. 2007). This might be due in part to the effort required to protect handover boundaries from perturbations. Nurses perform a lot of interactional work to maintain handover boundaries, which interferes with their own participation in the handover. While such work helps maintain the integrity of the handover, other participants may be distracted by the perturbation.

By nature, handovers interrupt other activities. As time goes by, those activities impinge on the handover, creating perturbations. This consumes attentional and interactional resources of handover participants as they attempt to manage the perturbations ad hoc. Procedures for preemptively reducing perturbations may therefore make handovers more focused and possibly shorter. Enacting such procedures may be supported by hospital guidelines, but may also ultimately hinge on handover participants themselves deciding how to best protect boundaries – given that they themselves are likely the commonest source of perturbations. The distinction between a perturbation and an interruption highlights the active nature of boundary management work nurses do and contributes towards a better understanding of the complexity of interruptions in medical settings (Hopkinson & Jennings 2013).

### Limitations

The local context (Switzerland) and the small sample (four units for quantitative analyses, one unit for qualitative analyses) might reduce generalizability of our findings.

## Conclusions

- Handover perturbations are frequent and may endanger continuity of care
- Nurses themselves are the most frequent sources of perturbations
- Perturbations are recognized by nurses and acted on, both verbally and non‐verbally, to maintain the integrity of handover boundaries
- Qualitative analyses revealed two main components of boundary management: division of labour and multimodality
- The importance of handover boundary management should be acknowledged in training and practice
- Furthermore, as perturbations can have an impact on continuity of care, hospital policies should encourage their minimization and all professionals should be trained to respect their boundaries
- More research is needed on management of perturbations and interruptions to do justice to the complexity of the phenomena

## Conflict of interest

No conflict of interest has been declared by the authors.

## Author contributions

All authors have agreed on the final version and meet at least one of the following criteria [recommended by the ICMJE (http://www.icmje.org/recommendations/)]:

- substantial contributions to conception and design, acquisition of data, or analysis and interpretation of data;
- drafting the article or revising it critically for important intellectual content.
